# Evaluation of Radiation Response in CoCrFeCuNi High-Entropy Alloys

**DOI:** 10.3390/e20110835

**Published:** 2018-10-31

**Authors:** Yang Wang, Kun Zhang, Yihui Feng, Yansen Li, Weiqi Tang, Bingchen Wei

**Affiliations:** 1 Key Laboratory of Microgravity (National Microgravity Laboratory), Institute of Mechanics, Chinese Academy of Sciences, Beijing 100190, China; 2 School of Engineering Science, University of Chinese Academy of Sciences, Beijing 100049, China; 3 State Key Laboratory of Nonlinear Mechanics, Institute of Mechanics, Chinese Academy of Sciences, Beijing 100190, China

**Keywords:** high-entropy alloy, ion irradiation, hardening behavior, volume swelling

## Abstract

CoCrFeCuNi high-entropy alloys (HEAs) prepared by arc melting were irradiated with a 100 keV He^+^ ion beam. Volume swelling and hardening induced by irradiation were evaluated. When the dose reached 5.0 × 10^17^ ions/cm^2^, the Cu-rich phases exhibited more severe volume swelling compared with the matrix phases. This result indicated that the Cu-rich phases were favorable sites for the nucleation and gathering of He bubbles. X-ray diffraction indicated that all diffraction peak intensities decreased regularly. This reduction suggested loosening of the irradiated layer, thereby reducing crystallinity, under He^+^ ion irradiation. The Nix-Gao model was used to fit the measured hardness in order to obtain a hardness value *H*_0_ that excludes the indentation size effect. At ion doses of 2.5 × 10^17^ ions/cm^2^ and 5.0 × 10^17^ ions/cm^2^, the HEAs showed obvious hardening, which could be attributed to the formation of large amounts of irradiation defects. At the ion dose of 1.0 × 10^18^ ions/cm^2^, hardening was reduced, owing to the exfoliation of the original irradiation layer, combined with recovery induced by long-term thermal spike. This study is important to explore the potential uses of HEAs under extreme irradiation conditions.

## 1. Introduction

High-entropy alloys (HEAs) have recently drawn increased interest because of their distinct compositions, microstructures, and flexible properties. In contrast to conventional alloys, HEAs are composed of more than five principal elements at equal or nearly equal atomic percentages (at.%). HEAs exerts four primary effects: (1) high-entropy effect; (2) sluggish diffusion effect; (3) severe lattice distortion effect; and (4) cocktail effect [1,2,3,4]. These effects render HEAs more likely to form a simple solid-solution structure rather than an intermetallic compound, which confers distinct properties on HEAs, including high fatigue strength [5], high hardness [6], good abrasion resistance and corrosion resistance, high breaking strength at low temperatures [7,8,9,10], and good softening resistance at elevated temperatures [11]. The use of HEAs as structural materials under extreme environments has been proposed owing to their desirable mechanical properties and thermodynamic stability. 

Previous studies on the irradiation effects of HEAs have mostly focused on irradiation resistance and phase stability. Zhang et al. [12] evaluated the effects of irradiation on Al_x_CoCrFeNi (x = 0.1, 0.75, and 1.5) HEAs under 3 MeV Au^+^ ion irradiation at room temperature. Results indicated that compared with conventional nuclear materials, single-phase HEAs based on the face-centered cubic (FCC) structure in the Al_x_CoCrFeNi system showed improved radiation resistance; in addition, volume swelling in the Al_x_CoCrFeNi alloys in the ascending order was FCC < FCC + BCC < BCC (body-centered cubic). Yang et al. [13] recently reported that with temperature increased from 523 K to 923 K, the irradiation-induced defect density of Al_0.1_CoCrFeNi HEAs decreased, whereas the size of the defect increased. Meanwhile, irradiation led to Ni and Co enrichment as well as Fe, Cr, and Al depletion in dislocation loops and dislocation regions. T. Nagase. et al. [14] showed that as-sputtered CoCrFeMnNi HEAs maintained good irradiation resistance within a wide temperature range from 298 to 773 K without grain coarsening under fast electron irradiation. Jin et al. studied a Ni-based multicomponent alloy under 3 MeV Ni ion irradiation at 773 K [15,16] and found that irradiation-induced volume swelling decreased with an increase in the number of elements in the disordered solid solution under identical irradiation conditions. This finding suggested that irradiation-induced volume swelling was also strongly affected by compositional complexity. 

In the last decade, FeCoNi-based HEAs, as one of the successful HEAs, have attracted more and more attention, especially because of its mechanical properties and microstructure evolution [17]. M. Klimova et al. [18] studied the microstructure and mechanical properties evolution of the Al-, C-containing CoCrFeNiMn-type high-entropy alloy during cold rolling. They reported that rolling resulted in an increase in strength and a decrease in ductility of the Al-, C-containing CoCrFeNiMn-type alloy. Feng et al. [19] found that the short range order is positive between Al-Al, Al-Si, Si-Si pairs and negative between Ni-Al, Co-Si, Fe-Co, Ni-Si, and Fe-Si pairs, which leads to an increase in the elastic modulus by sacrificing ductility and isotropy. In addition, the appropriate doping of Y_2_O_3_ as a reinforcement phase in CoCrFeMnNi HEAs could increase both the room temperature tensile strength and the wear-resistance [20].

With regard to the irradiation response of HEAs, there are not many researches on the evolution of mechanical properties of FeCoNi-based HEAs after irradiation. Meanwhile, most studies focused on the effect of composition on irradiation-induced swelling and vertical inhomogeneity of radiation damage, and the difference in irradiation response between the phase and phase boundary has rarely been reported. Whether lateral inhomogeneity of irradiation response exists in the HEAs, especially in single-phase FCC structure-based HEAs, has yet to be determined. In the present study, a typical single-phase FCC structure-based CoCrFeCuNi HEA was selected, and the key objective is to determine the irradiation response exerted by He^+^ ion irradiation. The present study provides an initial examination of the fundamental irradiation behavior of an HEA material, thus offering an insight into the potential of this family of materials for application under extreme environments.

## 2. Materials and Methods 

CoCrFeCuNi HEAs with a diameter of 5 mm and a length of 100 mm were prepared by arc melting a mixture of pure metal (purity > 99.9% wt %) in a Ti-gettered high-purity argon atmosphere. These ingots were remelted at least four times to prevent chemical heterogeneity and were eventually drop-cast into a copper mold. The cast bar was cut into thin pieces, each with a thickness of 2 mm, and then mechanically polished to a mirror finish. Irradiation experiments were conducted in a BNU-400 kV electrostatic accelerator in a test chamber, applying pressure near or below 10^−5^ torr. The polished samples were irradiated with a 100 keV He^+^ ion beam at fluences of 2.5 × 10^17^ ions/cm^2^, 5.0 × 10^17^ ions/cm^2^, and 1.0 × 10^18^ ions/cm^2^ at a normal angle at room temperature. 

**Figure 1:**
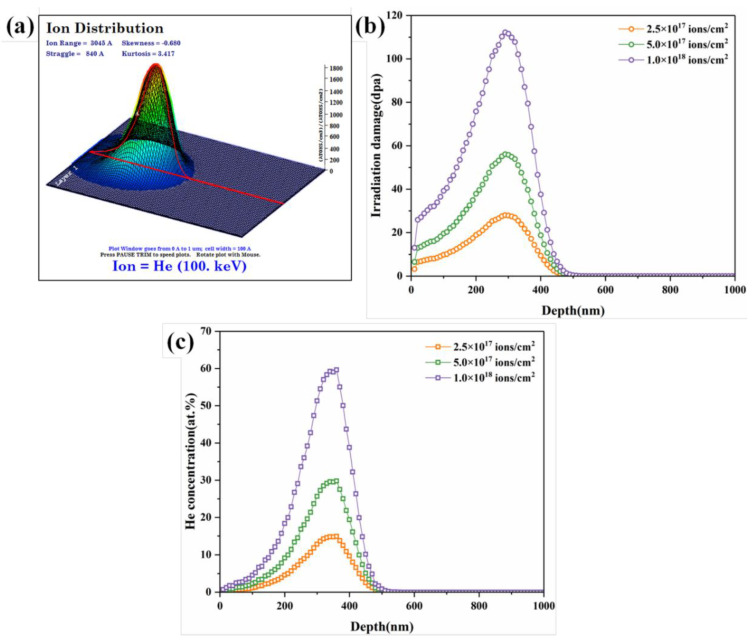
SRIM simulations of CoCrFeCuNi HEAs irradiated with 100 keV He^+^ ions. (**a**) Ion distribution; (**b**) irradiation damage (dpa); and (**c**) concentration of He atoms with depth.

Figure 1 shows the results for ion distribution, irradiation damage (displacement per atom or dpa), and concentration of He atoms with the increases in depth calculated using the Ion Distribution and Quick Calculation of Damage mode in the SRIM (Stopping and Range of Ions in Matter) 2008 code [21]. As shown in Figure 1a, when a large number of incident ions enter the HEAs, they form a spatial influence area rather than a specific path owing to collision cascade. The total number of ions is 99,999, and the effective depth of ion influence is ~560 nm. The dpa is determined by the sum of the predicted Co, Cr, Cu, Fe, and Ni vacancy concentrations and recoil events within 0 nm to ~560 nm from the surface. According to Figure 1b,c, the three curves in each figure linearly change with increased ion doses, implying that the dpa and concentration of He atoms are positively correlated with the total doses of incident ions. Equations (1) and (2) present the method used to extract the irradiation damage (total vacancies produced/atom = dpa) and He atom concentration (in at.%) from SRIM output files [21].

(1) $$\overset{\mathrm{vacancy}.\mathrm{txt}}{\overbrace{\left(\frac{vacancies}{ions\times Å}\right)}}\times \left(\frac{10^{8}\left(\frac{Å}{cm}\right)\times \mathrm{Fluence}\left(\frac{ions}{cm^{2}}\right)}{8.705\times 10^{22}\left(\frac{atoms}{cm^{3}}\right)}\right)=\mathrm{dpa},$$

(2) $$\overset{\mathrm{range}.\mathrm{txt}}{\overbrace{\left(\frac{atoms/cm^{3}}{atoms/cm^{2}}\right)}}\times \left(\frac{\mathrm{Fluence}\left(\frac{ions}{cm^{2}}\right)}{8.705\times 10^{22}\left(\frac{atoms}{cm^{3}}\right)}\right)\times 100=\left(\frac{ions}{atom}\right)\times 100=\mathrm{at}\%\;\mathrm{ion},$$

In addition, the number of atomic displacements reached the peak at a depth of approximately 290 nm, which corresponded to the projected range of the He^+^ ions in CoCrFeCuNi HEAs. The peak concentrations of the He atoms were approximately 14.9%, 29.8%, and 59.6% under different doses.

The phase structures of pristine and irradiated samples were analyzed by slow-scan X-Ray diffraction (XRD, Philips PW 1050 diffractometer with CuKα radiation, Amsterdam, Netherlands) at 45 kV voltage, 0.01 step size, and 200 mA current for phase analysis with improved accuracy. The microstructure and compositions of pristine and irradiated samples were analyzed by scanning electron microscopy (SEM, HITACHI S-4800, Tokyo, Japan) taken at a working voltage of 20.0 kV and a working distance of 14.3 and 15.0 mm, with energy-dispersive X-ray spectroscopy (EDX, HORIBA X-Max, Kyoto, Japan). 

Nanoindentation experiments on pristine and irradiated samples were conducted using the Agilent Technologies Nano Indenter G200 with a stand Berkovich diamond indenter at room temperature (29.0 °C). The hardness measurement parameter included a strain rate of ~0.05 s^−1^, frequency of ~45 Hz, and harmonic displacement of ~2.0 nm. Each nanoindentation test ran to a maximum of 1000 nm into the indented surface, and all hardness measurement positions were in the matrix phases. Specifically, the sample irradiated at fluences of up to 1.0 × 10^18^ ions/cm^2^, the hardness measurement positions were located apart from the exfoliation of the damaged areas, that is, on the remaining part underneath. The geometry of the tip was standardized from the indentation on fused silica with the same indentation depth, and each sample was subjected to 5 indents with a space wider than 50 μm.

## 3. Results

### 3.1. Microstructural Characterization

**Figure 2:**
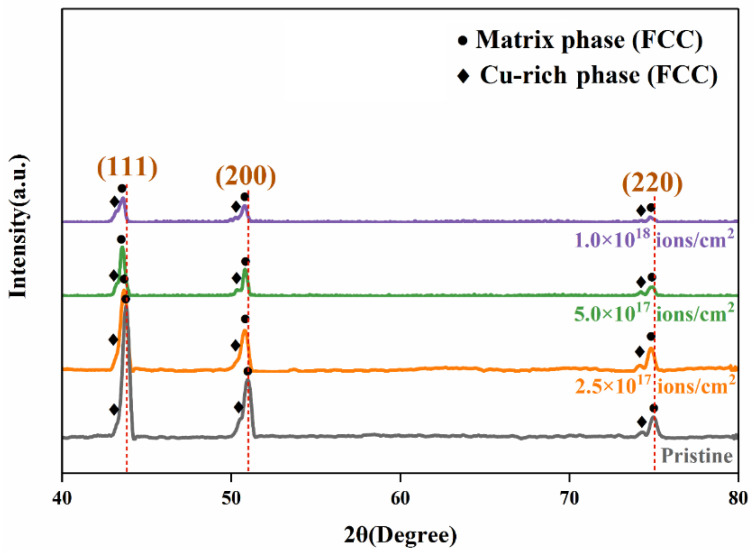
XRD patterns of pristine and He^+^ ion-irradiated CoCrFeCuNi HEAs at different doses.

The XRD patterns of the pristine and irradiated CoCrFeCuNi HEA samples at different doses are presented in Figure 2. The figure reveals that the samples remained fully crystalline, and the diffraction peaks of CoCrFeCuNi HEAs in both pristine and irradiated samples at 43°, 50°, and 74° are from the typical FCC phases corresponding to (111), (200), and (220) lattice planes, respectively. These findings are similar to those in previous studies [22,23]. Figure 2 shows that no obvious phase decomposition occurs under any irradiation condition, which is consistent with high configurational entropy playing a significant role in single-phase stability [24]. A closer examination reveals that each major peak contains two slightly separated peaks. The left peak of the major split peaks is attributed to the Cu-rich phases, whereas the right peak is attributed to the matrix phases. These findings are consistent with the following EDX results. The separated major peaks are hardly differentiated at low angles because no significant difference in average atomic radius between the Cu-rich phases and matrix phases in CoCrFeCuNi HEA is indicated. Moreover, the diffraction peaks of the irradiated HEAs shift to the left, meaning a slight lattice expansion happens. Meanwhile, the intensity of the diffraction peaks decreases with the increasing of irradiation dose, owing to the increased concentration of defects in the near surface of HEAs.

Irradiation has been known to enhance diffusion in solids by creating defects, such as vacancies and interstitials. These defects fill the irradiated layer with voids, eventually leading to a decrease in crystallinity. Irradiation-induced defects generally lead to structural damage in the HEAs, such as cluster of small defects and dislocation loops [25]. According to Makinson et al. [26], the peak diffraction intensity of X-rays depends on the concentration of defects in a material. Meanwhile, peak intensity decreases with an increase in defect concentration, and vice versa. In the present study, the defect concentration induced by He^+^ ion irradiation increases with an increase in irradiation dose of up to 1.0 × 10^18^ ions/cm^2^, leading to a decrease in peak intensity. Moreover, the low solubility of helium in CoCrFeCuNi HEAs leads to its precipitation into bubbles and voids [27], further decreasing the peak intensities.

### 3.2. Surface Topography

**Figure 3:**
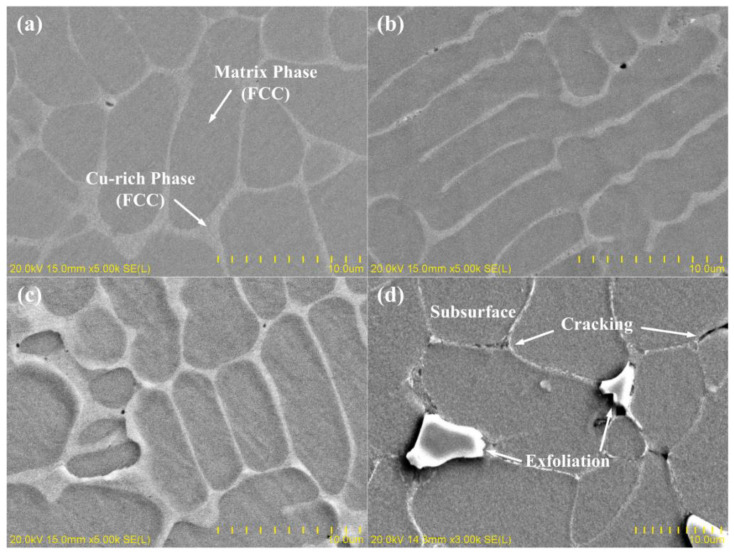
SEM images of CoCrFeCuNi HEAs. (**a**) Pristine and (**b**–**d**) He^+^ ions irradiated at fluences of 2.5 × 10^17^ ions/cm^2^, 5.0 × 10^17^ ions/cm^2^, and 1.0 × 10^18^ ions/cm^2^.

**Figure 4:**
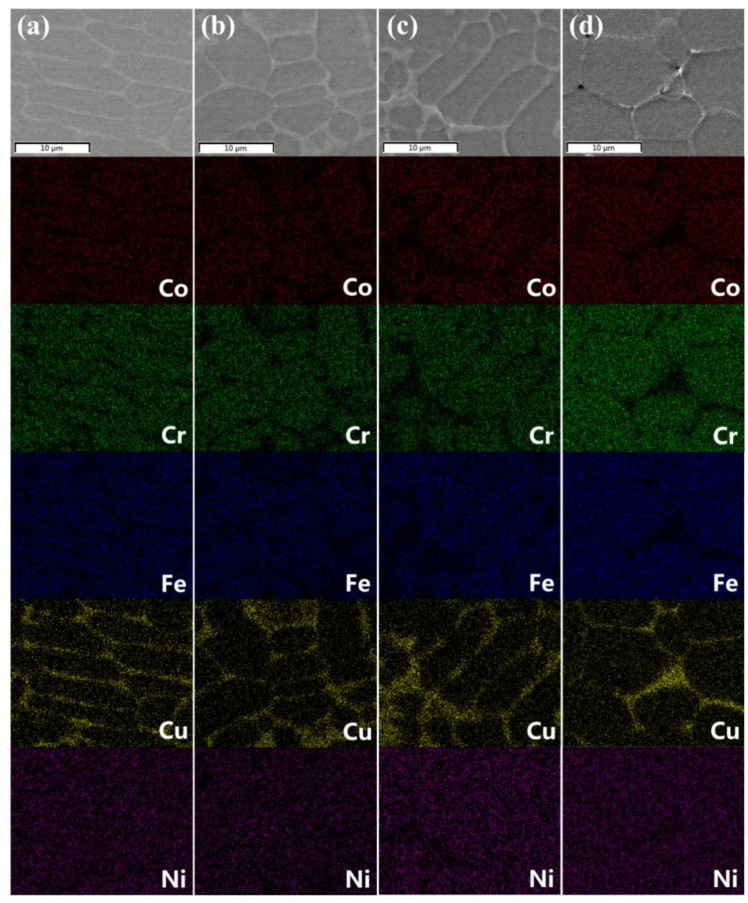
Energy-dispersive X-ray spectroscopy (EDX) maps showing elemental distribution of CoCrFeCuNi HEAs. (**a**) Pristine and (**b**–**d**) He^+^ ions irradiated at fluences of 2.5 × 10^17^ ions/cm^2^, 5.0 × 10^17^ ions/cm^2^, and 1.0 × 10^18^ ions/cm^2^.

The SEM images reveal the surface morphology of pristine and irradiated CoCrFeCuNi HEAs at different irradiation doses, as shown in Figure 3a–d. Table 1 shows the components of different regions in the microstructures of pristine and irradiated CoCrFeCuNi alloys estimated by EDX spectroscopy. The analysis was conducted on five pristine and five irradiated regions randomly chosen from the samples. The EDX maps showing the elemental distribution of Cr, Co, Fe, Cu, and Ni on the HEAs surface at different doses, are shown in Figure 4a–d, respectively. The EDX maps show matrix phases enriched with Cr, Fe, Co, and Ni, as well as Cu-rich phases at the phase boundaries with fair amounts of Ni, and no obvious diffusion occurs in the HEA components after irradiation, implying that the composition of HEA is stable after irradiation to some extent. Nevertheless, the distribution of the elements is very homogeneous in both the matrix phases and Cu-rich phases. Moreover, both the matrix phases and Cu-rich phases consist of only one simple phase, which is consistent with previous studies [28]. According to Figure 3b–d, when the irradiation dose increases to 5.0 × 10^17^ ions/cm^2^, the volume swelling of the Cu-rich phases markedly increases in severity. Meanwhile, no significant change is observed in the matrix phases, suggesting the inhomogeneity of radiation resistance on the surface perpendicular to the ion incident direction. The evolution of volume swelling after irradiation is discussed in the subsequent section.

However, at a dose of 1.0 × 10^18^ ions/cm^2^, cracking and exfoliation occur, and the subsurface becomes visible after the surface flakes off. Wei et al. [29] reported a similar occurrence on Cu_48_Zr_48_Al_4_ bulk metallic glass composites induced by He^+^ ion irradiation at the same dose. When high-energy He^+^ ions are bombarded at a target material, most ions take electrons from the matrix to form helium atoms, which can cause distortion, elastic rebound, and stress in the lattice [30]. With increasing irradiation fluence, irradiation-induced helium gas pressure develops inside the matrix. At a fluence of 1.0 × 10^18^ ions/cm^2^, excessive pressure of the helium gas was developed inside the irradiated area, which eventually led to cracking and exfoliation [31,32], as shown in Figure 3d.

### 3.3. Mechanical Behavior

**Figure 5:**
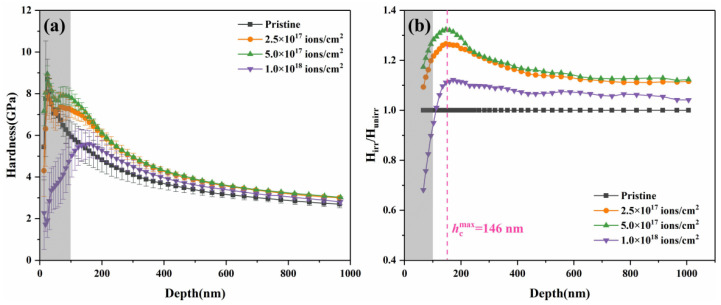
(**a**) Typical depth profiles of nanoindentation hardness and (**b**) dependence of the H_irr_/H_unirr_ ratio on the indentation depth of pristine and He+ ion-irradiated CoCrFeCuNi HEAs at different irradiation doses.

A nanoindentation test was conducted to reveal the average microhardness of the HEA surface and thus investigate the irradiation-induced hardening behavior. Figure 5 shows typical (a) depth profiles of nanoindentation hardness and (b) dependence of the H_irr_/H_unirr_ ratio on the indentation depth of pristine and irradiated CoCrFeCuNi HEAs at different irradiation doses. The irradiated region and substrate region are consistent with the dpa profiles (see Figure 1b). Figure 5a shows that the pristine samples decrease in hardness with increasing indent depth at the indentation depth of h > 50 nm. Such depth-dependent hardness behavior is regarded as the indentation size effect (ISE) [33]. By contrast, hardness increases with increasing indent depth at the depth of *h* < 50 nm is considered as reverse ISE, which is usually caused by uncertainty factors from the surface and the geometrical shape of the Berkovich indenter. Therefore, to exclude uncertainty factors from the surface, we ignored the data in regions shallower than 100 nm in this study. Compared with pristine samples, all irradiated samples exhibited different degrees of hardening at *h* > 100 nm. To more clearly characterize the degree of increment, the dependence of the H_irr_/H_unirr_ ratio on the indentation depth of all samples is exhibited in Figure 5b. Notably, the normalized nanoindentation hardness reaches a peak with an increase in indentation depth. The same peak position on all curves is located at a depth of ~146 nm (the dotted transition line). For the shallower region before the transition line, H_irr_/H_unirr_ increases with an increase in indentation depth, which is similar to the results for other metals [25] irradiated with similar ions. However, for the deeper region after the transition line, H_irr_/H_unirr_ decreases with an increase in indentation depth. 

An irradiation-induced hardened layer forms in the HEAs after He^+^ ion implantation. When indented into the sample surface, the hemispheric influence zone beneath the indenter can reach 4 to 10 times the indentation depth. As the indenter presses deeper, if the radius of the influence zone is less than the thickness of the hardened layer, the damage grade effect is exerted, and the hardening ratio increases; otherwise the softer substrate effect is exerted, and the hardening ratio decreases [34].

**Figure 6:**
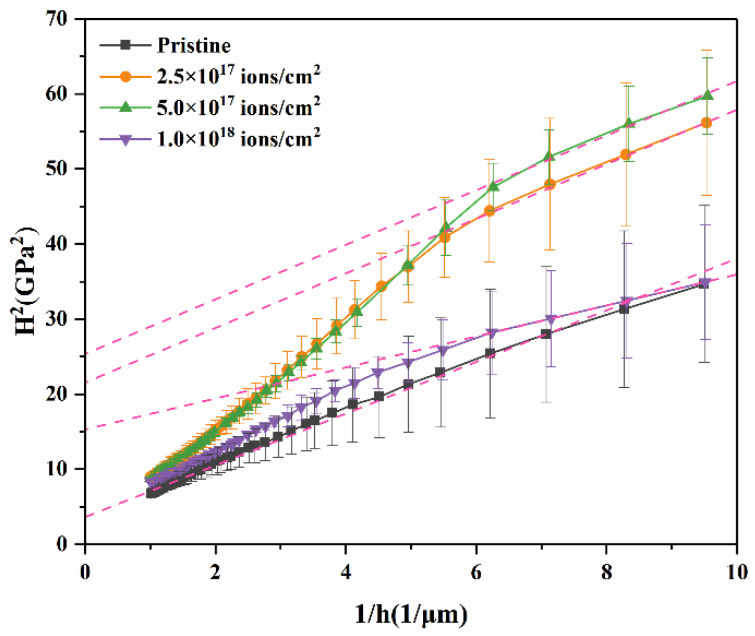
Plot of *H*^2^ versus 1/*h* for the pristine and irradiated HEAs at different doses.

To explain the ISE, Nix and Gao [35] developed a model based on the concept of geometrically necessary dislocations. This model predicts the hardness-depth profile by using the following equation.

(3) $$H=H_{0}{\left(1+\frac{h^{*}}{h}\right)}^{1/2},$$

where *H* is the hardness, *h* is the indentation depth, *H*_0_ is the hardness at an infinite depth (i.e., macroscopic hardness), and h* is the length that characterizes the depth dependence of hardness for a given material and indenter geometry, which depends on the material and shape of the indenter tip (i.e., statistically stored dislocation density) [36]. To aid the discussion, the hardness data are plotted as *H*^2^ versus 1/*h* in Figure 6. *H*_0_ is the square root of the intercept for the linear fitting of the hardness data in the near-surface region. The value of *H*_0_ excludes the size effect and can be used as a parameter to characterize the hardening effect in HEAs under irradiation.

In Figure 6, the pristine sample shows good linearity in the range of 100 nm < *h* < 1000 nm. Regardless, the irradiated samples exhibit bilinearity with a shoulder at the depth of about 150 nm consisting of the depth of the transition line, (see Figure 6). Table 2 lists *H*_0_ and h* for the pristine samples and irradiated samples calculated by least squares fitting of hardness data in the 100 nm < *h* < 1000 nm range according to Equation (3) [37].

The data in Table 2 shows that the calculated *H*_0_ first increases and then decreases with an increase in irradiation dose, which is consistent with the hardness results. The calculated h^*^ decreases systematically, implying a smaller size effect during irradiation, which is consistent with previous studies [37].

## 4. Discussion

**Figure 7:**
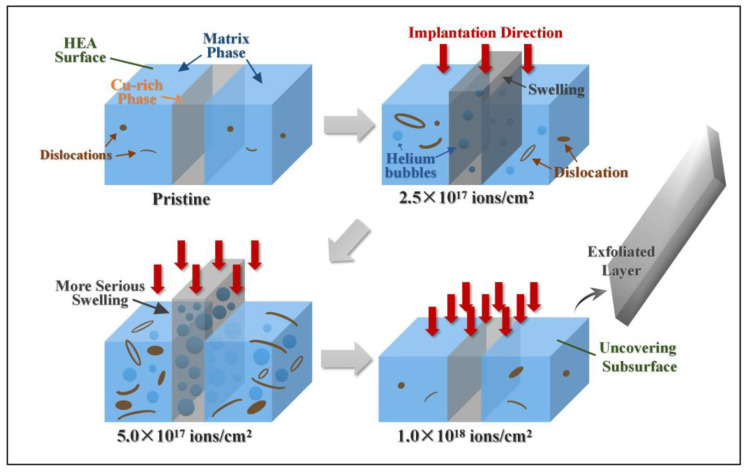
Schematic of radiation response in CoCrFeCuNi HEAs.

The schematic in Figure 7 illustrates the swelling effect, as well as the hardening behavior, of Cu-rich phases during He^+^ irradiation. With regard to the formation of He bubbles, nuclear loss-induced collisional damage, such as dislocations, voids, and interfaces, act as sinks for point defects. Dislocations can partly absorb more interstitials than vacancies. A large number of vacancies are consequently generated in the HEAs, and vacancy clusters grow and trap He to form He bubbles. For CoCrFeCuNi HEA, the Cu-rich phases appeared as defect-rich phase boundary regions in the matrix phases, similar to grain boundary, which are also favorable nucleation sites of He bubbles [38]. With the ion dose increasing to 5.0 × 10^17^ ions/cm^2^, the He concentration increasingly rises and reaches near-saturation [39] in the matrix phases. Thus, He bubbles tend to migrate and gather toward the Cu-rich phases, resulting in the increased severity of volume swelling, as shown in Figure 7. We also reported that the irradiation response is inhomogeneous along the ion incident direction because of a perpendicular local shear stress [40]. Cu-rich phases may act as local low-stress regions because of the more loosely packed He atoms. He bubbles may thus preferentially migrate toward the Cu-rich phases to unload the stress, resulting in the increased severity of volume swelling [41,42]. This factor can be potentially influence the volume swelling of Cu-rich phases.

At ion doses ranging from 2.5 × 10^17^ ions/cm^2^ to 5.0 × 10^17^ ions/cm^2^, HEAs exhibit marked hardening behavior, which can be attributed to obstacles to dislocation glide, proposed by Orowan and Seeger [43,44]. In general, irradiation-induced defect clusters and/or dislocation loops, as well as He bubbles, pin dislocation lines and impede dislocation glide, causing HEAs to harden [45]. However, as previously mentioned, as the irradiation dose increases, numerous He bubbles migrate and gather toward the Cu-rich phases to form cavities, which may induce swelling and softening of the Cu-rich phases. While the irradiation-induced dislocations continue to increase in the matrix phases, the overall result for the HEA ultimately reflects the hardening effect.

When the ion dose increases to 1.0 × 10^18^ ions/cm^2^, the hardness of the HEA slightly decreases. To avoid surface irregular interference, we selected the flatter subsurface for hardness testing. Consistent with the SEM micrograph in Figure 3d, at the highest dose, the uncovering of the subsurface after exfoliation corresponds to a new irradiation plane, except that it is only affected by diminished irradiation effects, and therefore the hardness decreases. In addition, recovery [46,47,48] might occur during irradiation. The thermal-spike effect during long-term irradiation can lead to annealing of defects and annihilation of helium bubbles, thus reducing the hardening effect. We hypothesize that the weakened irradiation effect on the uncovering subsurface, combined with recovery, causes softening of the HEA.

## 5. Conclusions

CoCrFeCuNi HEAs prepared by arc melting were irradiated with a 100 keV He^+^ ion beam at fluences of 2.5 × 10^17^ ions/cm^2^, 5.0 × 10^17^ ions/cm^2^, and 1.0 × 10^18^ ions/cm^2^ at room temperature. XRD results proved that all diffraction peak intensities along the (111), (200), and (220) lattice planes decreased regularly, indicating that the irradiated layer was filled with voids, leading to a decrease in crystallinity. When the irradiation dose increased to 5.0 × 10^17^ ions/cm^2^, the irradiation-induced swelling became increasingly severe in the Cu-rich phases, whereas no significant change occurred in the matrix phases. This difference suggested that the degree of swelling varied between the two phases under the same irradiation condition. This finding contributed to the migration and gathering of the He bubble-induced noncompact structure and the lateral inhomogeneity of radiation damage. At a higher dose, 1.0 × 10^18^ ions/cm^2^, cracking and exfoliation occurred revealing the subsurface after the surface flaked off mainly from the excessive pressure of the He gas. Nanoindentaion was performed to indicate that hardening induced by He^+^ ion irradiation occurred in both irradiated samples. The depth-dependent hardness behavior was explained by the Nix-Gao model. At ion doses of 2.5 × 10^17^ ions/cm^2^ and 5.0 × 10^17^ ions/cm^2^, the HEAs exhibited an obvious hardening behavior, which can be explained by dislocation-dominated hardening effect. When the ion dose reached 1.0 × 10^18^ ions/cm^2^, the hardening effect decreased probably because of the weakened irradiation effect on the uncovered subsurface, combined with the long-term thermal-spike effect-induced recovery.

## Figures and Tables

**Table 1:** Components of different regions in the microstructure of CoCrFeCuNi HEAs (at. %).

Condition	Region	Co	Cr	Fe	Cu	Ni
Nominal	―	20	20	20	20	20
Pristine	Matrix Phase	22.87 ± 0.52	23.08 ± 0.24	22.67 ± 0.46	10.75 ± 0.62	20.46 ± 0.50
	Cu-rich Phase	5.60 ± 0.57	6.26 ± 0.85	6.53 ± 0.62	68.86 ± 2.15	12.76 ± 0.48
2.5 × 10^17^ ions/cm^2^	Matrix Phase	22.83 ± 0.47	23.41 ± 0.19	22.59 ± 0.32	10.64 ± 0.11	20.38 ± 0.31
	Cu-rich Phase	6.29 ± 0.71	7.11 ± 0.49	6.92 ± 0.91	69.39 ± 3.18	10.29 ± 0.62
5.0 × 10^17^ ions/cm^2^	Matrix Phase	22.66 ± 0.65	23.23 ± 0.38	23.06 ± 0.43	10.61 ± 0.36	20.43 ± 0.59
	Cu-rich Phase	4.69 ± 0.32	5.19 ± 1.24	5.19 ± 0.92	74.09 ± 3.06	10.85 ± 0.72
1.0 × 10^1^^8^ ions/cm^2^	Matrix phase	23.55 ± 0.12	23.30 ± 0.18	22.85 ± 0.75	10.68 ± 0.29	19.62 ± 0.61
	Cu-rich phase	9.84 ± 0.99	11.33 ± 1.50	11.00 ± 0.92	51.66 ± 3.93	16.19 ± 0.77

**Table 2:** Calculated *H*_0_ and h* based on the Nix–Gao model.

Materials	Irradiation Doses (ions/cm^2^)	*H*_0_ (GPa)	h* (nm)
CoCrCuFeNi HEAs	0	1.948	902
	2.5 × 10^17^	4.779	152
	5.0 × 10^17^	5.063	137
	1.0 × 10^18^	3.934	130
